# Age‐Specific Trends in Carcinoma Incidence Among Adolescents and Young Adults in the United States, 1975–2021

**DOI:** 10.1002/cam4.71627

**Published:** 2026-02-20

**Authors:** Adrian D. Aguilar, Satya Batna, Rebecca D. Kehm

**Affiliations:** ^1^ Department of Epidemiology, Mailman School of Public Health Columbia University New York USA; ^2^ Herbert Irving Comprehensive Cancer Center Columbia University Medical Center New York USA

**Keywords:** adolescents and young adults, cancer, carcinomas, time trends

## Abstract

**Introduction:**

The incidence of carcinomas among adolescents and young adults (AYAs; ages 15–39) is rising. However, most research treats AYAs as a single group, potentially obscuring important age‐ and sex‐specific trends. This study examined long‐term trends in carcinoma incidence among AYAs in the U.S., overall and by 5 year age groups.

**Methods:**

We conducted a population‐based, retrospective time‐series analysis using data from eight U.S. cancer registries in the Surveillance, Epidemiology and End Results database. The study included 128,255 AYAs diagnosed with malignant carcinoma between 1975–2021. Average annual percent change (AAPC) in incidence rates was estimated using Joinpoint regression, stratified by age, sex, and carcinoma subtype.

**Results:**

A total of 128,255 individuals, aged 15–39, who were diagnosed with carcinoma between 1975–2021; 77.5% of AYA carcinoma cases occurred in females. The overall incidence of carcinomas increased by 0.61% (CI: 0.43–0.80) per year in AYAs from 1975 to 2021, with similar increases observed in females (AAPC: 0.68%; 95% CI, 0.50–0.87) and males (AAPC: 0.71%; 95% CI, 0.45–0.97). When stratified by age group, overall carcinoma incidence increased in each 5‐year group; the largest relative increase was in those aged 15–19 (AAPC: 1.66%; 95% CI, 0.71–2.62) and the smallest was in those aged 35–39 (AAPC: 0.48%; 95% CI, 0.22–0.74).

**Conclusions:**

Carcinoma incidence is increasing in the AYA population within each 5‐year age group, including among adolescents, a group in which these cancers have historically been rare. These findings highlight the urgent need to investigate the causes of this increase to guide targeted prevention and early detection efforts.

## Graphical abstract

This study analyzed long‐term trends in carcinoma incidence among U.S. adolescents and young adults (ages 15–39) using cancer registry data from 1975 to 2021. The study found that carcinoma incidence increased steadily over time in both males and females and across all 5 year age groups, with the largest relative rise among adolescents aged 15–19. These findings point to the need for research on the causes of this increase to inform prevention and early detection strategies.

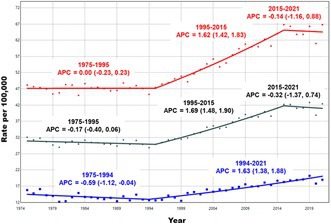

## 1 Introduction

Cancer is often thought of as a disease for older adults [1], with a median age at diagnosis of 66 years [2]. However, adolescents and young adults (AYAs), representing individuals aged 15–39 years, are also affected by cancer and they are an often overlooked population [3, 4]. Recent global estimates indicate that in 2019, there were approximately 1.19 million new cancer cases and 396,000 deaths among individuals aged 15–39 [4]. This represents a substantial rise in what is known as early‐onset cancer, with global incidence increasing by 79.1% between 1990–2019 [5]. While accidents are the primary cause of death among adolescents, cancer ranks as the foremost cause of disease‐related fatalities in this demographic [6]. Despite the heavy toll that cancer takes on the AYA population, there remains a considerable gap in our understanding of the epidemiology surrounding cancers in this age group.

While there has been an increase in AYA cancer incidence in the past decade, historically, most oncology research has focused on either adult cancers or childhood cancers, leaving cancer occurring in AYAs understudied [7]. This oversight has hindered the development of tailored diagnosis, treatment, and monitoring guidelines specifically for AYAs. Consequently, the management of AYA patients often relies on frameworks borrowed from pediatric and adult oncology, which do not adequately address the unique biological, social, and economic challenges faced by this population [8].

One of the major challenges in understanding AYA cancers is the unique and heterogeneous distribution of cancer types within this population. Among adolescents aged 15–19 years, the most frequently diagnosed cancers include lymphomas, leukemias, sarcomas, and brain tumors [9]. In young adults, aged 20–29, melanoma, thyroid cancer, and testicular cancer become more prevalent [9]. As the AYA population ages into their 30s, carcinomas such as breast and colorectal occur with increasing frequency [9]. Given this shifting distribution of cancer types, studies that treat AYAs as a single, homogeneous population may overlook important age‐specific differences in cancer incidence trends.

A growing body of evidence indicates that the incidence of several early‐onset carcinomas, including colorectal, breast, and thyroid cancers, is increasing in both the U.S. and globally [10, 11, 12, 13]. However, most studies have examined these trends by treating the AYA population as a single group, without disaggregating trends by narrower age subgroups. As a result, it is unclear whether the rising incidence is primarily occurring among older AYAs or also affecting younger individuals, in whom these cancers are typically rare. Addressing this gap is essential for identifying age‐specific incidence patterns, uncovering contributing risk factors, and informing targeted prevention and early detection strategies within the diverse AYA population. This effort is especially urgent given the persistent underrepresentation of AYAs in carcinoma‐focused clinical trials [14], limiting the development of evidence‐based, age‐appropriate treatment and care approaches.

In this study, we addressed this gap by using U.S. population‐based data to analyze long‐term trends in AYA carcinoma incidence rates from 1975 to 2021. We assessed carcinoma incidence trends overall and for the most common carcinoma subtypes, stratified by 5 year age groups and sex.

## 2 Methods

### 2.1 Data Sources

We used the National Cancer Institute's Surveillance, Epidemiology, and End Results (SEER) 8 database to assess trends in AYA carcinoma incidence rates from 1975 to 2021. SEER 8 covers approximately 8.3% of the U.S. population (based on the 2020 census) and includes cancer registries from the Atlanta metropolitan area, Connecticut, Hawaii, Iowa, New Mexico, the Seattle‐Puget Sound region, the San Francisco‐Oakland metropolitan area, and Utah [15]. Although SEER 8 has the smallest geographic coverage among the SEER databases, it includes the longest span of available data, making it the optimal dataset for examining long‐term cancer incidence trends. We received approval to use de‐identified cancer incidence data through a SEER Public Use Research Data Agreement. The study was exempt from ethical review and complies with Strengthening the Reporting of Observational Studies in Epidemiology (STROBE) reporting guidelines [16].

### 2.2 Study Population

In this population‐based time‐series analysis, we included individuals aged 15–39 years who were diagnosed with a malignant carcinoma (defined as code 9 in the SEER AYA Site Recode 2020 Revision [17]) between 1975–2021 (*n* = 128,255). We used SEER*Stat software to calculate annual age‐adjusted incidence rates (standardized to the 2000 U.S. population) and 95% confidence intervals (CIs), applying the Tiwari et al. (2006) modification for CI estimation [18]. We calculated incidence rates for the AYA population overall and stratified by 5 year age groups, including adolescents (aged 15–19) and young adults (aged 20–24, 25–29, 30–34, and 35–39). We calculated incidence rates for all carcinomas combined and for specific carcinoma subtypes based on SEER AYA Site Recode 2020 Revision classifications [17]. These subtypes included thyroid carcinoma (9.1), other carcinomas of the head and neck (9.2), gastrointestinal tract carcinomas (9.3), carcinomas of the lung, bronchus, and trachea (9.4), skin carcinomas (9.5), breast carcinomas (9.6), carcinomas of genital sites excluding ovary and testis (9.7), urinary tract carcinomas (9.8), and other invasive carcinomas (9.9). All incidence rates were further stratified by sex (female and male).

### 2.3 Statistical Analysis

We used Joinpoint software (version 5.4.0 [19]), from the National Cancer Institute to evaluate trends in overall carcinoma incidence rates (all subtypes combined) among AYAs from 1975 to 2021. Analyses were conducted for the entire AYA population (ages 15–39) and further stratified by 5 year age groups and sex. We also examined trends for carcinoma subtypes, restricting analyses to those with sufficient sample size, defined as having no years with zero case counts in any age group. This excluded skin carcinomas from the overall AYA analysis (ages 15–39), and only thyroid and gastrointestinal tract carcinomas were included in the age‐stratified analyses.

Joinpoint regression models trends by fitting a series of connected straight lines on a logarithmic scale and uses multiple permutation testing to identify inflection points (joinpoints) where the trend changes [20, 21, 22]. We fit joinpoint regression models that allowed for 0 to 6 joinpoints, with a minimum of 5 observations between adjacent inflection points. We used the weighted Bayesian Information Criterion method to select the best‐fitting log‐linear model. We calculated the annual percent change (APC) for each joinpoint segment and corresponding 95% CIs using the parametric method [23]. To summarize trends over the full study period (1975–2021), we calculated the average annual percent change (AAPC) as a weighted average of the APCs across all segments, with weights proportional to the length of each segment [23]. Statistical significance of the AAPC was evaluated using a two‐sided *t*‐test, with a *p* < 0.05. AAPC values greater than zero reflect increasing trends, while AAPC values less than zero reflect decreasing trends.

## 3 Results

### 3.1 Sample Characteristics

A total of 128,255 individuals, aged 15–39, who were diagnosed with carcinoma between 1975–2021 were included in the analysis, including 28,814 male (22.5%) and 99,441 female (77.5%) participants. The number of carcinoma cases increased with age across the AYA population, rising from 2924 cases (2.3% of all AYA carcinoma cases) in the 15–19 age group to 63,448 cases (49.5%) in the 35–39 age group (Table 1). Overall and in females, thyroid carcinoma was the most common subtype in individuals aged 15–29, while breast carcinoma was most common in those aged 30–39. Among males, thyroid carcinoma was the most common subtype in individuals aged 15–24, whereas gastrointestinal tract carcinomas were most common in those aged 25–39. The number of cases for each gastrointestinal carcinoma subtype is provided in (Table S1).

**TABLE 1:** Distribution of carcinoma cases among adolescents and young adults, by age group and sex, SEER 8 Registries, 1975–2021.

Sex	Age group, No. (%) of cases				
Carcinoma type	15–19	20–24	25–29	30–34	35–39
Both sexes combined					
Thyroid	1607 (55.0%)	3751 (49.8%)	6534 (35.6%)	8651 (24.0%)	9968 (15.7%)
Head & Neck	309 (10.6%)	549 (7.3%)	980 (5.3%)	1625 (4.5%)	2789 (4.4%)
Gastrointestinal Tract	532 (18.2%)	1196 (15.9%)	2586 (14.1%)	5169 (14.4%)	9927 (15.6%)
Lung, Bronchus, Trachea	95 (3.2%)	189 (2.5%)	393 (2.1%)	1074 (3.0%)	2826 (4.5%)
Skin	13 (0.4%)	28 (0.4%)	34 (0.2%)	74 (0.2%)	126 (0.2%)
Breast	36 (1.2%)	506 (6.7%)	3435 (18.7%)	10,850 (30.1%)	24,319 (38.3%)
Genital (excluding Ovary & Testis)	84 (2.9%)	727 (9.7%)	3030 (16.5%)	5924 (16.5%)	8317 (13.1%)
Urinary Tract	180 (6.2%)	397 (5.3%)	1007 (5.5%)	2024 (5.6%)	4109 (6.5%)
Other	68 (2.3%)	182 (2.4%)	365 (2.0%)	603 (1.7%)	1067 (1.7%)
Overall	2924 (100%)	7525 (100%)	18,364 (100%)	35,994 (100%)	63,448 (100%)
Females					
Thyroid	1318 (64.6%)	3133 (56.1%)	5384 (37.4%)	7030 (24.7%)	7880 (16.1%)
Head & Neck	155 (7.6%)	270 (4.8%)	487 (3.4%)	673 (2.4%)	1035 (2.1%)
Gastrointestinal Tract	266 (13.0%)	599 (10.7%)	1267 (8.8%)	2390 (8.4%)	4474 (9.1%)
Lung, Bronchus, Trachea	51 (2.5%)	102 (1.8%)	204 (1.4%)	521 (1.8%)	1337 (2.7%)
Skin	5 (0.2%)	14 (0.3%)	19 (0.1%)	27 (0.1%)	66 (0.1%)
Breast	36 (1.8%)	504 (9.0%)	3428 (23.8%)	10,824 (38.1%)	24,256 (49.5%)
Genital (excluding Ovary & Testis)	79 (3.9%)	718 (12.9%)	3015 (21.0%)	5873 (20.7%)	8061 (16.4%)
Urinary Tract	89 (4.4%)	151 (2.7%)	410 (2.8%)	760 (2.7%)	1392 (2.8%)
Other	41 (2.0%)	89 (1.6%)	176 (1.2%)	319 (1.1%)	513 (1.0%)
Overall	2040 (100%)	5580 (100%)	14,390 (100%)	28,417 (100%)	49,014 (100%)
Males					
Thyroid	289 (32.7%)	618 (31.8%)	1150 (28.9%)	1621 (21.4%)	2088 (14.5%)
Head & Neck	154 (17.4%)	279 (14.3%)	493 (12.4%)	952 (12.6%)	1754 (12.2%)
Gastrointestinal Tract	266 (30.1%)	597 (30.7%)	1319 (33.2%)	2779 (36.7%)	5453 (37.8%)
Lung, Bronchus, Trachea	44 (5.0%)	87 (4.5%)	189 (4.8%)	553 (7.3%)	1489 (10.3%)
Skin	8 (0.9%)	14 (0.7%)	15 (0.4%)	47 (0.6%)	60 (0.4%)
Breast	0 (0.0%)	2 (0.1%)	7 (0.2%)	26 (0.3%)	63 (0.4%)
Genital (excluding Ovary & Testis)	5 (0.6%)	9 (0.5%)	15 (0.4%)	51 (0.7%)	256 (1.8%)
Urinary Tract	91 (10.3%)	246 (12.6%)	597 (15.0%)	1264 (16.7%)	2717 (18.9%)
Other	27 (3.1%)	93 (4.8%)	189 (4.8%)	284 (3.7%)	554 (3.8%)
Overall	884 (100%)	1945 (100%)	3974 (100%)	7577 (100%)	14,434 (100%)

**FIGURE 1:**
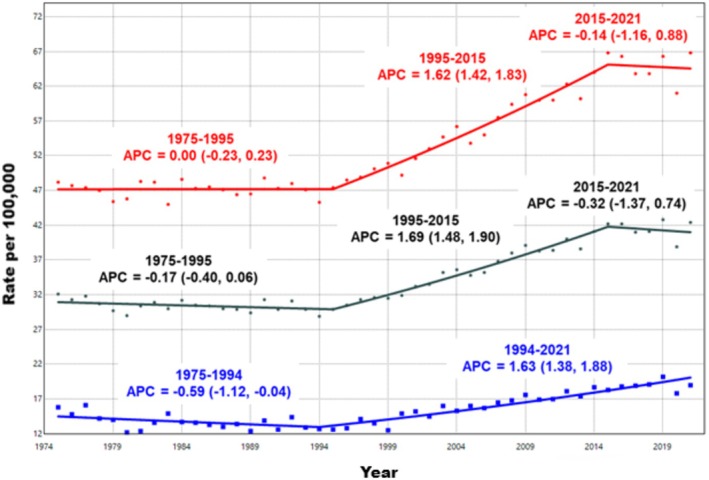
Trends in overall carcinoma incidence rates among adolescents and young adults by sex, SEER 8 Registries, 1975–2021. Age‐adjusted incidence trends of carcinomas (includes all diagnoses coded as 9 based on the SEER AYA Site Recode 2020 Revision) among adolescents and young adults (ages 15–39) in the SEER 8 Registries from 1975 to 2021. Trends for both sexes combined are shown in black, females in red, and males in blue. Rates are per 100,000 population and age‐adjusted to the 2000 U.S. standard population (19 age groups, Census P25‐1130). Trends are modeled using Joinpoint regression with a maximum of six Joinpoints, requiring a minimum of 5 years per segment and between each segment and the ends of the data series. The annual percent change (APC) for each segment is displayed above the corresponding trend line, with 95% confidence intervals shown in parentheses.

Among AYAs aged 15–39, carcinoma incidence statistically significantly increased by an average of 0.61% (95% CI: 0.43, 0.80) per year from 1975 to 2021 (Table 2). Similar upward trends were observed in both females (AAPC: 0.68%, 95% CI: 0.50, 0.87) and males (AAPC: 0.71%, 95% CI: 0.45, 0.97). The increase in AYA carcinomas began in the mid‐1990s, with rates stabilizing among females between 2015–2021, while continuing to increase in men (Figure 1).

**TABLE 2:** Average annual percent change in carcinoma incidence rates among adolescents and young adults, ages 15–39 years, overall and by sex, SEER 8 Registries, 1975–2021.

Carcinoma subtype	Both sexes combined	Females	Males
	AAPC (95% CI)	AAPC (95% CI)	AAPC (95% CI)
Thyroid	1.86 (1.41, 2.30)	2.07 (1.59, 2.55)	0.89 (−0.36, 2.16)
Head and neck	0.64 (−1.29, 2.61)	0.70 (−0.34, 1.74)	0.11 (−1.25, 1.48)
Gastrointestinal tract	1.64 (1.32, 1.96)	1.77 (1.31, 2.23)	1.40 (1.00, 1.80)
Lung, bronchus, trachea	‐1.96 (−2.18, −1.74)	−1.72 (−2.08, −1.36)	−2.57 (−2.85, −2.29)
Skin	NA	NA	NA
Breast	0.45 (0.18, 0.73)	0.49 (0.19, 0.78)	NA
Genital (excluding ovary & testis)	−0.67 (−1.13, −0.20)	−0.65 (−1.10, −0.20)	NA
Urinary tract	1.18 (0.53, 1.83)	1.99 (1.62, 2.36)	0.67 (−0.21, 1.57)
Other	−1.47 (−2.22, −0.72)	−2.71 (−4.13, −1.28)	−0.77 (−1.56, 0.03)
Overall	0.61 (0.43, 0.80)	0.68 (0.50, 0.87)	0.71 (0.45, 0.97)

Abbreviations: AAPC, average annual percent change; CI, confidence interval; NA, not available due to small case counts.

When stratified by cancer subtype, statistically significant increases in incidence were observed for thyroid carcinomas (AAPC: 1.86%, 95% CI: 1.41, 2.30), gastrointestinal tract carcinomas (AAPC: 1.64%, 95% CI: 1.32, 1.96), breast carcinomas (AAPC: 0.45%, 95% CI: 0.18, 0.73), and urinary tract carcinomas (AAPC: 1.18%, 95% CI: 0.53, 1.83). For each of these subtypes, the AAPC was higher in females compared to males. Notably, from 1975 to 2021, the annual rate of increase in incidence among females was more than twice that of males for both thyroid carcinoma (2.07% versus 0.89%) and urinary tract carcinomas (1.99% versus 0.67%). The increase in breast carcinoma incidence among females (AAPC: 0.49%, 95% CI: 0.19, 0.78) also contributed to the sex differences, as male breast carcinoma was extremely rare (*n* = 98).

From 1975 to 2021, there were statistically significant declines in the incidence of lung, bronchus, and trachea carcinomas (AAPC: −1.96, 95% CI: −2.18, −1.74), genital (excluding ovary and testis) carcinomas (AAPC: −0.67, 95% CI: −1.13, −0.20), and other carcinoma types (AAPC: −1.47, 95% CI: −2.22, −0.72). The decline in lung, bronchus, and trachea carcinomas was more pronounced in males compared to females, while the decrease in genital carcinomas was observed only in females.

### 3.2 Age‐Specific Carcinoma Incidence Trends in the AYA Population

When stratified by age, overall carcinoma incidence (all subtypes combined) statistically significantly increased across all AYA age groups from 1975 to 2021 (Table 3). The largest increase was observed in the 15–19 age group (AAPC: 1.66%, 95% CI: 0.71, 2.62), while the smallest increase occurred in the 35–39 age group (AAPC: 0.48%, 95% CI: 0.22, 0.74). When further stratified by sex, males had a higher rate of increase in overall carcinoma incidence in the two youngest age groups [15, 16, 17, 18, 19, 20, 21, 22, 23, 24, 25, 26, 27, 28, 29, 30, 31, 32, 33, 34], whereas females experienced a higher rate of increase in the three older age groups. The most pronounced increase in carcinoma incidence was observed among adolescent males aged 15–19, who experienced a 2.08% (95% CI: 1.53, 2.63) annual increase from 1975 to 2021.

**TABLE 3:** Average annual percent change in carcinoma incidence trends among adolescents and young adults by age group and sex, SEER 8 Registries, 1975–2021.

Carcinoma subtype	15–19	20–24	25–29	30–34	35–39
Sex	AAPC (95% CI)	AAPC (95% CI)	AAPC (95% CI)	AAPC (95% CI)	AAPC (95% CI)
Overall					
Both sexes	1.66 (0.71, 2.62)	0.96 (0.30, 1.63)	0.50 (0.11, 0.90)	0.61 (0.28, 0.93)	0.48 (0.22, 0.74)
Females	1.56 (0.36, 2.77)	0.97 (0.26, 1.68)	0.67 (0.24, 1.10)	0.65 (0.31, 0.99)	0.63 (0.44, 0.82)
Males	2.08 (1.53, 2.63)	1.54 (0.81, 2.28)	0.52 (−0.37, 1.43)	0.56 (−0.04, 1.16)	0.37 (0.05, 0.70)
Thyroid					
Both sexes	1.70 (0.42, 2.99)	2.07 (1.77, 2.38)	1.20 (0.40, 1.90)	1.59 (0.90, 2.28)	2.60 (2.04, 3.17)
Females	1.60 (0.06, 3.16)	2.11 (1.78, 2.43)	1.30 (0.56, 2.05)	1.98 (1.15, 2.81)	2.79 (2.11, 3.47)
Males	2.40 (1.52, 3.29)	1.97 (0.80, 3.16)	−0.63 (−3.24, 2.06)	0.53 (−0.57, 1.65)	1.51 (−0.34, 3.40)
Gastrointestinal tract					
Both sexes	4.12 (2.41, 5.86)	2.82 (1.43, 4.23)	1.87 (0.97, 2.77)	1.59 (1.00, 2.19)	1.16 (0.79, 1.54)
Females	3.55 (1.17, 5.98)	3.01 (0.82, 5.24)	2.80 (1.83, 3.79)	1.54 (0.65, 2.43)	1.27 (0.71, 1.83)
Males	3.47 (1.72, 5.26)	3.00 (2.33, 3.68)	1.64 (0.61, 2.67)	1.74 (1.42, 2.07)	0.72 (−0.17, 1.62)

Abbreviations: AAPC, average annual percent change; CI, confidence interval.

**FIGURE 2:**
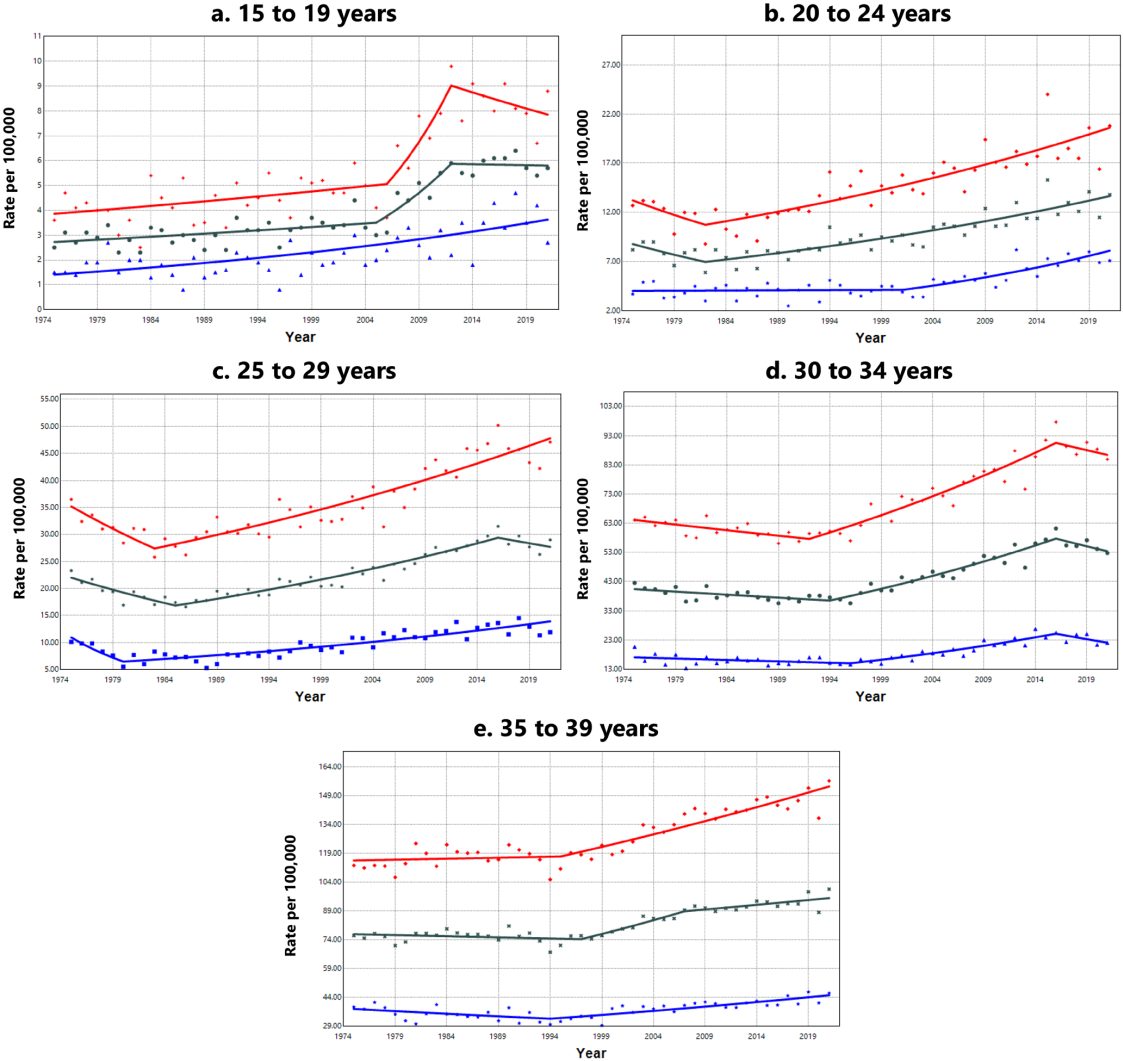
Trends in overall carcinoma incidence rates among adolescents and young adults by age group and sex, SEER 8 Registries, 1975–2021. Age‐adjusted incidence trends of carcinomas (includes all diagnoses coded as 9 based on the SEER AYA Site Recode 2020 Revision) among adolescents and young adults (ages 15–39) in the SEER 8 Registries from 1975 to 2021. Trends for both sexes combined are shown in black, females in red, and males in blue. Rates are per 100,000 population and age‐adjusted to the 2000 U.S. standard population (19 age groups, Census P25‐1130). Trends are modeled using Joinpoint regression with a maximum of six Joinpoints, requiring a minimum of 5 years per segment and between each segment and the ends of the data series. The annual percent change for each segment is available in (Table S2).

The 15–19 age group was the only group in which incidence rates were statistically significantly increasing since the beginning of the study period in 1975 (Figure 2). Among males in this age group, rates rose steadily throughout the entire study period. In contrast, females aged 15–19 experienced a sharp increase in incidence beginning in 2006, which plateaued in 2012. In the older AYA age groups, the onset of rising carcinoma incidence occurred progressively later, starting in 1982 for ages 20–24 and as late as 1997 for ages 35–39, when combining both sexes. When stratified by sex, this increase began earlier in females (1982) than in males (2001) in the 20–24 year age group. In the three older age groups, sex differences in the shape of the segmented trends were less pronounced. However, across all age groups, females consistently had higher absolute carcinoma incidence rates than males. APC values for each joinpoint segment are provided in (Table S2), which highlight a notable spike in incidence rates among 15–19 year‐old females between 2006 to 2012.

### 3.3 Thyroid Carcinomas

From 1975 to 2021, thyroid carcinoma incidence statistically significantly increased across all AYA age groups when analyzing both sexes combined, as well as among females alone. Among females, the largest increase was observed in the oldest age group, with an AAPC of 2.79% (95% CI: 2.11, 3.47). In contrast, among males, significant increases in thyroid carcinoma incidence were observed only in the two youngest age groups, with the largest increase occurring among 15–19 year‐old males (AAPC: 2.40%, 95% CI: 1.52, 3.29). APC values for each joinpoint segment of age‐specific thyroid carcinoma incidence trends are provided in (Table S3).

### 3.4 Gastrointestinal Carcinomas

The incidence of gastrointestinal tract carcinomas statistically significantly increased across all sexes and AYA age groups, except for in 35–39 year‐old males. In both females and males, the largest increases were observed in the youngest age group, with AAPCs of 3.55% (95% CI: 1.17, 5.98) for females and 3.47% (95% CI: 1.72, 5.26) for males. The magnitude of the increase declined progressively with age in both sexes. APC values for each joinpoint segment of age‐specific gastrointestinal tract carcinoma incidence trends are provided in (Table S4).

## 4 Discussion

This study examined long‐term trends in carcinoma incidence rates among U.S. AYAs aged 15–39 in the U.S. from 1975 to 2021. While previous studies have evaluated cancer incidence trends in the AYA population as a whole, this study is among the first to analyze carcinoma incidence across narrower 5 year age subgroups, providing more nuanced insights into age‐specific incidence trends. Our findings confirm a sustained increase in carcinoma incidence over time within the AYA population overall, largely driven by the rise in thyroid, gastrointestinal, urinary tract, and breast carcinomas. This is consistent with previous research documenting increases in early‐onset cancers, typically defined as those diagnosed before age 50 [10, 11, 12, 13, 24, 25, 26]. This study also offers new evidence that the rising trend in carcinoma incidence is occurring across the age range of AYAs, including among adolescents, a group in which carcinomas have historically been rare. In fact, individuals aged 15–19 experienced the largest relative increase in incidence between 1975–2021 among both males and females. Although adolescents have experienced the greatest relative increase in carcinomas over time, the absolute number of carcinoma cases remains significantly higher in the older AYA age groups. In fact, nearly half of all AYA carcinomas were diagnosed in the oldest 35–39 age group, indicating that the overall burden of carcinomas continues to disproportionately impact older age groups within the AYA population.

We also identified differences in carcinoma incidence between females and males in the AYA population. Notably, more than three‐quarters of AYA carcinomas occurred in females, primarily due to the higher incidence of thyroid and breast carcinomas among females compared to males. There were also sex differences in carcinoma incidence trends over the study period. In the 15–19 age group, carcinoma incidence rates increased steadily from 1975 to 2021 among males. In contrast, among 15–19 year‐old females, a sharp increase in carcinoma incidence (overall and for thyroid and gastrointestinal tract carcinomas) occurred between 2006–2012. Segmented incidence trends were more consistent between sexes in the older AYA age groups, suggesting that distinct factors may be contributing to the sex‐specific trends observed during adolescence.

The underlying causes of the rise in carcinoma incidence among AYAs remain unclear. Some of the increase in incidence may be due to improved surveillance and earlier detection over time. For example, the increased use of diagnostic imaging techniques, such as ultrasound and computed tomography (CT), may have contributed to the rising incidence of certain carcinomas, particularly thyroid cancer, over time [11, 27, 28]. This may also help explain the higher rates of thyroid carcinoma in AYA females compared to males, as females are more likely to engage with the healthcare system and undergo diagnostic imaging [29]. In 2006, the American Thyroid Association issued guidelines recommending, for the first time, that thyroid nodules in children be evaluated using the same diagnostic approaches as in adults [30, 31]. This may have led to increased use of diagnostic ultrasonography and ultrasonography‐guided biopsies in the pediatric population, resulting in greater detection of thyroid cancers [32]. Notably, the timing of this guideline coincides with the sharp increase in thyroid carcinoma incidence observed specifically among females aged 15–19, supporting that changing medical practices may have directly influenced incidence trends in the AYA population. Changes in diagnostic classifications over time may also have contributed to the observed increase in AYA carcinomas. For example, the reclassification of appendix carcinoids as malignant tumors in the first revision of the third edition of the International Classification of Diseases for Oncology (ICD‐O‐3), which was released in 2013, has been shown to account for part of the documented rise in early‐onset gastrointestinal carcinomas over time [33].

However, improved detection and changes in diagnostic classification alone are unlikely to fully account for the rise in AYA carcinoma incidence over time. Mounting evidence suggests that factors such as dietary changes, endocrine‐disrupting chemicals, increased use of antibiotics, and rising obesity rates may also contribute to this trend [11, 12, 13, 14, 15, 16, 17, 18, 19, 20, 21, 22, 23, 24, 34, 35, 36]. For gastrointestinal carcinomas, there is growing interest in the role of the gut microbiome, which can be altered by lifestyle and environmental factors such as obesity, physical inactivity, diet, and antibiotic use [37]. Emerging evidence suggests that microbial‐host interactions are stronger in early‐onset colorectal cancer compared to late‐onset colorectal cancer, supporting a potential role of the microbiome in the rising incidence of AYA gastrointestinal carcinomas [38]. Obesity is a well‐established risk factor for several cancer types, including gastrointestinal carcinomas and certain urinary tract carcinomas like kidney cancer [39]. Notably, the rise in obesity in the U.S., which began in the late 1970s, closely parallels the increasing incidence of some early‐onset cancers, such as colorectal cancer [40]. However, it is important to note that obesity is not an established risk factor for all of the carcinoma subtypes that have been increasing over time. Further, obesity is associated with lower premenopausal breast cancer risk [41], suggesting that additional risk factors contribute to the overall rise in AYA carcinomas.

Hormonal influences such as estrogen exposure, earlier onset of puberty, and reproductive patterns are known contributors to breast and thyroid cancer risk [42, 43, 44, 45], and may help explain the higher incidence of carcinomas among female AYAs compared to males. Additionally, mounting evidence suggests that environmental chemicals, particularly those with endocrine‐disrupting properties, may influence hormone‐related cancer risk [46, 47, 48]. For example, early‐life exposure to dichlorodiphenyltrichloroethane (DDT) has been associated with increased risk of premenopausal breast cancer [49]. Another example is the emerging evidence linking pre‐ and polyfluoroalkyl substances, a class of persistent endocrine‐disrupting chemicals introduced in the 1940s, to increased thyroid cancer risk [50]. As the number of chemicals in circulation continues to rise, with the Chemical Abstract Service Registry growing from 20 million to 156 million chemicals between 2002–2019 [51], further research on the role of environmental exposures in cancer etiology, particularly in the AYA population, is urgently needed.

This study has several key strengths. Most notably, it is among the first to disaggregate carcinoma incidence trends across 5 year AYA age groups, offering more precise insights than prior studies that treated AYAs as a single group. Additionally, the use of SEER data spanning from 1975 to 2021 allowed for the analysis of long‐term trends, and stratification by both sex and carcinoma subtype provided further insights into demographic‐specific incidence patterns that can inform targeted prevention and clinical strategies. However, this study also has limitations. Although SEER 8 offers the longest span of historical cancer incidence data, its limited geographic coverage reduces case counts and may restrict the generalizability of findings to other regions of the U.S. and international populations. Yet, our previous research supports that early‐onset cancer incidence trends observed in SEER 8 are consistent with those in broader SEER datasets like SEER 13 [10]. Additionally, because carcinomas are relatively rare in the AYA population, our analysis was limited to broad carcinoma categories, with only a few specific subtypes examined within individual age groups. We also did not stratify by other potentially important demographic factors, such as race and ethnicity, due to small case counts. Moreover, because SEER does not capture data on individual risk, factors such as obesity, lifestyle behaviors, or environmental exposures, we cannot draw conclusions about the underlying causes of the observed shift in AYA carcinoma incidence. Finally, because this study focused on incidence rates of AYA carcinomas incidence rates, we were unable to determine whether the observed increases reflect a true rise in disease burden or are partly due to earlier detection or improved diagnostic practices over time. Future research examining survival and mortality trends in AYA carcinomas will be critical to clarifying the underlying drivers of the increasing incidence. Nevertheless, this study offers insights that can guide future research and generate hypotheses aimed at uncovering the drivers of rising carcinoma rates in the AYA population.

In conclusion, this study provides new evidence that the rising incidence of AYA carcinomas spans the entire age range of this population, underscoring the urgent need to investigate the underlying causes of these trends. Past success such as the decline in lung carcinomas following reductions in smoking [52], and the decrease in female genital carcinomas after the introduction of the human papilloma virus vaccine [53], demonstrate that incidence trends can be reversed. These examples highlight the tremendous potential for targeted and population‐based strategies to reduce the growing burden of carcinomas in the AYA population.

## Author Contributions

**Adrian D. Aguilar:** conceptualization (supporting), formal analysis (lead), visualization (lead), writing – original draft (lead), writing – review and editing (equal). **Satya Batna:** data curation (supporting), formal analysis (supporting), visualization (supporting), writing – review and editing (equal). **Rebecca D. Kehm:** conceptualization (lead), data curation (lead), funding acquisition (lead), writing – original draft (supporting), writing – review and editing (equal).

## Funding

This work was supported by the National Cancer Institute, R00CA263024.

## Ethics Statement

The study used deidentified data and was exempt from ethical review.

## Conflicts of Interest

The authors declare no conflicts of interest.

## Supporting information


**Table S1:** Distribution of gastrointestinal tract carcinoma cases among adolescents and young adults, by age group and sex, SEER 8 Registries, 1975–2021.
**Table S2:**. Average annual percent change in overall carcinoma incidence trends among adolescents and young adults by age group and sex, SEER 8 Registries, 1975–2021.
**Table S3:**. Average annual percent change in thyroid carcinoma incidence trends among adolescents and young adults by age group and sex, SEER 8 Registries, 1975–2021.
**Table S4:**. Average annual percent change in gastrointestinal tract carcinoma incidence trends among adolescents and young adults by age group and sex, SEER 8 Registries, 1975–2021.

## Data Availability

The data that support the findings of this study are available from the corresponding author upon reasonable request.
