# Supplementary material for: Age‐Specific Trends in Carcinoma Incidence Among Adolescents and Young Adults in the United States, 1975–2021
Source: Cancer Med. 2026 Feb 20;15(3):e71627. doi: 10.1002/cam4.71627 (PMC12921416; doi:10.1002/cam4.71627)
Supplement: Supplementary file 1 — Table S1: Distribution of gastrointestinal tract carcinoma cases among adolescents and young adults, by age group and sex, SEER 8 Registries, 1975–2021. Table S2:. Average annual percent change in overall carcinoma incidence trends among adolescents and young adults by age group and sex, SEER 8 Registries, 1975–2021. Table S3:. Average annual percent change in thyroid carcinoma incidence trends among adolescents and young adults by age group and sex, SEER 8 Registries, 1975–2021. Table S4:. Average annual percent change in gastrointestinal tract carcinoma incidence trends among adolescents and young adults by age group and sex, SEER 8 Registries, 1975–2021. [file CAM4-15-e71627-s001.docx]

| **Supplementary Table S1.** Distribution of gastrointestinal tract carcinoma cases among adolescents and young adults, by age group and sex, SEER 8 Registries, 1975-2021 | | | | | |
| --- | --- | --- | --- | --- | --- |
| **Sex** | Age Group, No. (%) of cases | | | | |
| Gastrointestinal tract carcinoma subtype | 15-19 | 20-24 | 25-29 | 30-34 | 35-39 |
| **Both sexes combined** |  |  |  |  |  |
| 9.3.1 Carcinoma of the esophagus | 2 (0.4%) | 16 (1.3%) | 50 (1.9%) | 103 (2%) | 250 (2.5%) |
| 9.3.2 Carcinoma of the stomach | 24 (4.5%) | 109 (9.1%) | 269 (10.4%) | 602 (11.6%) | 1118 (11.3%) |
| 9.3.3 Carcinoma of the small intestine | 11 (2.1%) | 35 (2.9%) | 71 (2.7%) | 169 (3.3%) | 318 (3.2%) |
| 9.3.4 Carcinoma of the colon | 306 (57.5%) | 608 (50.8%) | 1175 (45.4%) | 2131 (41.2%) | 3854 (38.8%) |
| 9.3.5 Carcinoma of the rectum | 47 (8.8%) | 193 (16.1%) | 558 (21.6%) | 1151 (22.3%) | 2287 (23%) |
| 9.3.6 Carcinoma of the anus | 2 (0.4%) | 18 (1.5%) | 50 (1.9%) | 170 (3.3%) | 381 (3.8%) |
| 9.3.7 Carcinoma of the liver and intrahepatic bile ducts | 84 (15.8%) | 103 (8.6%) | 213 (8.2%) | 314 (6.1%) | 646 (6.5%) |
| 9.3.8 Carcinoma of the gallbladder or other extrahepatic biliary | 4 (0.8%) | 12 (1%) | 39 (1.5%) | 102 (2%) | 219 (2.2%) |
| 9.3.9 Carcinoma of the pancreas | 49 (9.2%) | 94 (7.9%) | 145 (5.6%) | 396 (7.7%) | 802 (8.1%) |
| 9.3.10 Other carcinoma of the gastrointestinal tract | 3 (0.6%) | 8 (0.7%) | 16 (0.6%) | 31 (0.6%) | 52 (0.5%) |
| *9.3 Carcinoma of gastrointestinal tract* | *532 (100%)* | *1196 (100%)* | *2586 (100%)* | *5169 (100%)* | *9927 (100%)* |
| **Females** |  |  |  |  |  |
| 9.3.1 Carcinoma of the esophagus | 0 (0%) | 3 (0.5%) | 15 (1.2%) | 13 (0.5%) | 40 (0.9%) |
| 9.3.2 Carcinoma of the stomach | 12 (4.5%) | 53 (8.8%) | 133 (10.5%) | 288 (12.1%) | 514 (11.5%) |
| 9.3.3 Carcinoma of the small intestine | 1 (0.4%) | 19 (3.2%) | 32 (2.5%) | 77 (3.2%) | 145 (3.2%) |
| 9.3.4 Carcinoma of the colon | 157 (59%) | 302 (50.4%) | 622 (49.1%) | 1044 (43.7%) | 1911 (42.7%) |
| 9.3.5 Carcinoma of the rectum | 22 (8.3%) | 92 (15.4%) | 264 (20.8%) | 543 (22.7%) | 1047 (23.4%) |
| 9.3.6 Carcinoma of the anus | 0 (0%) | 7 (1.2%) | 14 (1.1%) | 49 (2.1%) | 149 (3.3%) |
| 9.3.7 Carcinoma of the liver and intrahepatic bile ducts | 41 (15.4%) | 43 (7.2%) | 74 (5.8%) | 98 (4.1%) | 195 (4.4%) |
| 9.3.8 Carcinoma of the gallbladder or other extrahepatic biliary | 1 (0.4%) | 6 (1%) | 15 (1.2%) | 55 (2.3%) | 111 (2.5%) |
| 9.3.9 Carcinoma of the pancreas | 30 (11.3%) | 69 (11.5%) | 90 (7.1%) | 203 (8.5%) | 339 (7.6%) |
| 9.3.10 Other carcinoma of the gastrointestinal tract | 2 (0.8%) | 5 (0.8%) | 8 (0.6%) | 20 (0.8%) | 23 (0.5%) |
| *9.3 Carcinoma of gastrointestinal tract* | *266 (100%)* | *599 (100%)* | *1267 (100%)* | *2390 (100%)* | *4474 (100%)* |
| **Males** |  |  |  |  |  |
| 9.3.1 Carcinoma of the esophagus | 2 (0.8%) | 13 (2.2%) | 35 (2.7%) | 90 (3.2%) | 210 (3.9%) |
| 9.3.2 Carcinoma of the stomach | 12 (4.5%) | 56 (9.4%) | 136 (10.3%) | 314 (11.3%) | 604 (11.1%) |
| 9.3.3 Carcinoma of the small intestine | 10 (3.8%) | 16 (2.7%) | 39 (3%) | 92 (3.3%) | 173 (3.2%) |
| 9.3.4 Carcinoma of the colon | 149 (56%) | 306 (51.3%) | 553 (41.9%) | 1087 (39.1%) | 1943 (35.6%) |
| 9.3.5 Carcinoma of the rectum | 25 (9.4%) | 101 (16.9%) | 294 (22.3%) | 608 (21.9%) | 1240 (22.7%) |
| 9.3.6 Carcinoma of the anus | 2 (0.8%) | 11 (1.8%) | 36 (2.7%) | 121 (4.4%) | 232 (4.3%) |
| 9.3.7 Carcinoma of the liver and intrahepatic bile ducts | 43 (16.2%) | 60 (10.1%) | 139 (10.5%) | 216 (7.8%) | 451 (8.3%) |
| 9.3.8 Carcinoma of the gallbladder or other extrahepatic biliary | 3 (1.1%) | 6 (1%) | 24 (1.8%) | 47 (1.7%) | 108 (2%) |
| 9.3.9 Carcinoma of the pancreas | 19 (7.1%) | 25 (4.2%) | 55 (4.2%) | 193 (6.9%) | 463 (8.5%) |
| 9.3.10 Other carcinoma of the gastrointestinal tract | 1 (0.4%) | 3 (0.5%) | 8 (0.6%) | 11 (0.4%) | 29 (0.5%) |
| *9.3 Carcinoma of gastrointestinal tract* | *266 (100%)* | *597 (100%)* | *1319 (100%)* | *2779 (100%)* | *5453 (100%)* |

| **Supplementary Table S2.** Average annual percent change in overall carcinoma incidence trends among adolescents and young adults by age group and sex, SEER 8 Registries, 1975-2021 | | | | | | | | | |
| --- | --- | --- | --- | --- | --- | --- | --- | --- | --- |
| Sex | Segment 1 | |  | Segment 2 | | Segment 3 | | Segment 4 | |
| Age group | Timeframe | APC (95% CI) |  | Timeframe | APC (95% CI) | Timeframe | APC (95% CI) | Timeframe | APC (95% CI) |
| Both sexes |  |  |  |  |  |  |  |  |  |
| 15-19 | 1975-2005 | 0.84 (0.31, 1.38) |  | 2005-2012 | 7.68 (2.38, 13.26) | 2012-2021 | -0.14 (-2.57, 2.36) | - | - |
| 20-24 | 1975-1982 | -3.25 (-7.23, 0.89) |  | 1982-2021 | 1.74 (1.45, 2.03) | - | - | - | - |
| 25-29 | 1975-1985 | -2.64 (-3.80, -1.46) |  | 1985-2016 | 1.81 (1.60, 2.02) | 2016-2021 | -1.18 ( -3.72, 1.42) | - | - |
| 30-34 | 1975-1994 | -0.53 (-0.94, -0.13) |  | 1994-2016 | 2.10 (1.80, 2.40) | 2016-2021 | -1.55 (-3.78, 0.74) | - | - |
| 35-39 | 1975 -1997 | -0.16 (-0.43, 0.12) |  | 1997-2007 | 1.80 (0.90, 2.71) | 2007-2021 | 0.53 (0.11, 0.96) | - | - |
| Females |  |  |  |  |  |  |  |  |  |
| 15-19 | 1975-2006 | 0.88 (0.24, 1.52) |  | 2006-2012 | 10.13 (2.00, 18.91) | 2012-2021 | -1.53 (-4.30, 1.32) | - | - |
| 20-24 | 1975-1982 | -2.93 (-7.18, 1.51) |  | 1982-2021 | 1.68 (1.38, 1.99) | - | - | - | - |
| 25-29 | 1975-1983 | -2.65 (-4.48, -0.78) |  | 1983-2005 | 1.14 (0.71, 1.58) | 2005-2016 | 2.64 (1.49, 3.80) | 2016-2021 | -1.88 (-4.74,1.06) |
| 30-34 | 1975-1992 | -0.63 (-1.13, -0.12) |  | 1992-2016 | 1.90 (1.63, 2.17) | 2016 - 2021 | -0.93 (-3.27, 1.48) | - | - |
| 35-39 | 1975-1998 | 0.06 (-0.22, 0.34) |  | 1998-2007 | 1.85 (0.70, 3.01) | 2007-2021 | 0.57 (0.11, 1.02) | - | - |
| Males |  |  |  |  |  |  |  |  |  |
| 15-19 | 1975-2021 | 2.10 (1.50, 2.60) |  | - | - | - | - | - | - |
| 20-24 | 1975-2001 | 0.10 (-0.85, 1.07) |  | 2001-2021 | 3.44 (2.23, 4.66) | - | - | - | - |
| 25-29 | 1975-1980 | -9.97 (-17.02, -2.33) |  | 1980-2021 | 1.89 (1.58, 2.19) | - | - | - | - |
| 30-34 | 1975-1996 | -0.60 (-1.25, 0.05) |  | 1996-2016 | 2.61 (1.97, 3.26) | 2016-2021 | -2.60 (-6.67, 1.65) | - | - |
| 35-39 | 1975-1994 | -0.76 (-1.42, -0.08) |  | 1994-2021 | 1.18 (0.86, 1.50) | - | - | - | - |
| Abbreviations: AAPC, average annual percent change; CI, confidence interval | | | | | |  |  |  |  |

| **Supplementary Table S3.** Average annual percent change in thyroid carcinoma incidence trends among adolescents and young adults by age group and sex, SEER 8 Registries, 1975-2021 | | | | | | | | | |
| --- | --- | --- | --- | --- | --- | --- | --- | --- | --- |
| Sex | Segment 1 | |  | Segment 2 | | Segment 3 | | Segment 4 | |
| Age group | Timeframe | APC (95% CI) |  | Timeframe | APC (95% CI) | Timeframe | APC (95% CI) | Timeframe | APC (95% CI) |
| Both sexes |  |  |  |  |  |  |  |  |  |
| 15-19 | 1975-2005 | 1.26 (0.49, 2.03) |  | 2005-2012 | 9.12 (2.00, 16.73) | 2012-2021 | -2.31 (-5.43, 0.91) | - | - |
| 20-24 | 1975-2021 | 2.07 (1.77, 2.38) |  | - | - | - | - | - | - |
| 25-29 | 1975-1985 | -3.33 (-5.77, -0.82) |  | 1985-2013 | 3.91 (3.41, 4.41) | 2013-2021 | -2.42 (-4.67, -0.13) | - | - |
| 30-34 | 1975-1990 | -0.96 (-2.42, 0.53) |  | 1990-2015 | 4.73 (4.19, 5.26) | 2015-2021 | -4.66 (-7.67, -1.55) | - | - |
| 35-39 | 1975 -1997 | 1.91 (1.19, 2.64) |  | 1997-2003 | 8.99 (4.23, 13.96) | 2003 - 2011 | 4.47 (2.18, 6.81) | 2011 - 2021 | -1.12 (-2.27, 0.03) |
| Females |  |  |  |  |  |  |  |  |  |
| 15-19 | 1975-2006 | 1.61 (0.72, 2.51) |  | 2006-2012 | 10.10 (0.05, 21.16) | 2012-2021 | -3.76 (-7.39, 0.02) | - | - |
| 20-24 | 1975-2021 | 2.11 (1.78, 2.43) |  | - | - | - | - | - | - |
| 25-29 | 1975-1986 | -2.85 (-5.10, -0.54) |  | 1986-2013 | 4.13 (3.59, 4.67) | 2013-2021 | -2.24 (-4.52, 0.10) | - | - |
| 30-34 | 1975-1989 | -0.17 (-2.16, 1.85) |  | 1989-2015 | 4.65 (4.06, 5.26) | 2015-2021 | -4.22 (-7.65,-0.66) | - | - |
| 35-39 | 1975-1994 | 2.20 (0.99, 3.42) |  | 1994- 2010 | 6.45 (5.38, 7.53) | 2010-2021 | -1.34 (-2.63, -0.03) | - | - |
| Males |  |  |  |  |  |  |  |  |  |
| 15-19 | 1975-2021 | 2.40 (1.52, 3.29) |  | - | - | - | - | - | - |
| 20-24 | 1975-2003 | 0.12 (-1.29, 1.54) |  | 2003-2021 | 4.93 (2.74, 7.18) | - | - | - | - |
| 25-29 | 1975-1980 | -11.25 (-23.99, 3.62) |  | 1980-2016 | 2.91 (2.25, 3.58) | 2016-2021 | -6.69 (-16.57, 4.36) | - | - |
| 30-34 | 1975-1995 | -2.61 (-4.08, -1.12) |  | 1995-2014 | 6.30 (4.80, 7.83) | 2014-2021 | -5.40 (-9.78, -0.80) | - | - |
| 35-39 | 1975-1981 | -8.05 (-20.20, 5.94) |  | 1981-2021 | 3.03 (2.52, 3.54) | - | - | - | - |
| Abbreviations: AAPC, average annual percent change; CI, confidence interval | | | | | |  |  |  |  |

| **Supplementary Table S4.** Average annual percent change in gastrointestinal tract carcinoma incidence trends among adolescents and young adults by age group and sex, SEER 8 Registries, 1975-2021 | | | | | | | |
| --- | --- | --- | --- | --- | --- | --- | --- |
| Sex | Segment 1 | |  | Segment 2 | | Segment 3 | |
| Age group | Timeframe | APC (95% CI) |  | Timeframe | APC (95% CI) | Timeframe | APC (95% CI) |
| Both sexes |  |  |  |  |  |  |  |
| 15-19 | 1975-2010 | 2.13 (1.22, 3.04) |  | 2010-2016 | 22.64 (10.69, 35.88) | 2016-2021 | -2.04 (-9.15, 5.64) |
| 20-24 | 1975-1988 | -2.28 (-6.53, 2.17) |  | 1988- 2021 | 4.90 (4.00, 5.80) | - | - |
| 25-29 | 1975-1986 | -2.71 (-6.04, 0.74) |  | 1986 -2021 | 3.35 (2.86, 3.84) | - | - |
| 30-34 | 1975-1990 | -0.39 (-2.02, 1.26) |  | 1990-2021 | 2.57 (2.14, 3.00) | - | - |
| 35-39 | 1975 -1993 | -0.29 (-1.12, 0.55) |  | 1993-2021 | 2.11 (1.79, 2.43) | - | - |
| Females |  |  |  |  |  |  |  |
| 15-19 | 1975-2006 | -0.66 (-2.58, 1.30) |  | 2006-2016 | 20.86 (12.51, 29.83) | 2016-2021 | -1.67(-12.46, 10.45) |
| 20-24 | 1975-1985 | -5.54 (-14.08, 3.84) |  | 1985-2021 | 5.52 (4.43, 6.61) | - | - |
| 25-29 | 1975-2003 | 1.30 (0.07, 2.56) |  | 2003-2021 | 5.18 (3.49, 6.90) | - | - |
| 30-34 | 1975-1990 | -1.75 (-4.13, 0.70) |  | 1990-2021 | 3.17 (2.54, 3.81) | - | - |
| 35-39 | 1975-1995 | -0.62 (-1.71, 0.48) |  | 1995- 2021 | 2.75 (2.19, 3.30) | - | - |
| Males |  |  |  |  |  |  |  |
| 15-19 | 1975-2021 | 3.90 (3.01, 4.79) |  | - | - | - | - |
| 20-24 | 1975-2021 | 3.00 (2.33, 3.68) |  | - | - | - | - |
| 25-29 | 1975-1988 | -1.89 (-5.08, 1.41) |  | 1988-2021 | 3.06 (2.39, 3.73) | - | - |
| 30-34 | 1975-2021 | 1.74 (1.42, 2.07) |  | - | - | - | - |
| 35-39 | 1975-1981 | -4.05 (-10.74, 2.19) |  | 1981-2021 | 1.53 (1.26, 1.80) | - | - |
| Abbreviations: AAPC, average annual percent change; CI, confidence interval | | | | | |  |  |
